# Letter from the New Editor-in-Chief

**DOI:** 10.3390/cancers8010009

**Published:** 2016-01-06

**Authors:** Samuel C. Mok

**Affiliations:** Department of Gynecologic Oncology and Reproductive Medicine, The University of Texas MD Anderson Cancer Center, Houston, TX 77030, USA; scmok@mdanderson.org

Dear authors, reviewers, and readers of *Cancers*,

It gives me great pleasure to welcome you to the latest issue of *Cancers*, for which I now serve as Editor-in-Chief. First, thanks are due to my predecessor and the founding Editor-in-Chief, Dr. Robert Weiss. Over the past six years, he has done an outstanding job in shaping the journal to ensure its place as an online journal addressing both clinical and basic science issues related to cancer research. Citations of the journal are now available in PubMed and full text is available in PubMed central. Articles published in *Cancers* are also indexed by Embase, Scopus, and other databases. Dr. Weiss has always acted with the utmost academic integrity, which makes him a hard act to follow. I am very aware of the responsibilities that the Editor-in-Chief role entails, and I approach my new role with excitement. 

Under my leadership, the journal will continue its open access format, which will certainly evolve to ensure that the journal takes full advantage of the rapidly changing world of information and knowledge dissemination. The journal will continue to publish high-quality clinical, translational, and basic science research on cancer prevention, initiation, progression, and treatment, as well as other related topics. Peer review will remain a vital component of our assessment of submitted articles. Articles will be reviewed by editorial board members, who will pre-select high-quality and original articles before the articles are reviewed by at least two reviewers who are experts in the field of the research. This will ensure that all published articles are of the highest quality. The ultimate goal is to achieve high citation numbers and high impact for the journal.

I am fortunate to be supported by a highly effective team in the editorial office. The current group of Associate Editors work incredibly hard in the assessment and processing of submitted articles. A group of new Associate Editors who are experts in various fields of cancer research will be added to the editorial board, which will provide guidance for the journal.

As I assume the new role of Editor-in-Chief, I welcome input from our readers and contributors. Together with our outstanding team of editorial board members and highly effective editorial office team, I look forward to taking the journal to the next level. 

